# Thrombolytic Effects of Philippine Pit Viper (Trimeresurus flavomaculatus) Venom in Human Blood In Vitro and Ferric Chloride-Induced Cardiac Thrombosis on Swiss Webster Mice In Vivo

**DOI:** 10.7759/cureus.40856

**Published:** 2023-06-23

**Authors:** Aljeirou Alcachupas, Krisverlyn Bellosillo, Wynlee Rhm Catolico, Mark Cullen Davis, Alyssa Diaz, Yvette Karla Doyongan, Reczy Eduarte, Emerald Gersava, Mary Bernadette Intrepido, Maugri Grace Kristi Laluma, Candra Carmelli Lavalle, Jeffrey Millan

**Affiliations:** 1 College of Medicine, West Visayas State University, Iloilo City, PHL

**Keywords:** fibrinolysis, thrombosis, snake venom, clotting, thrombolysis, trimeresurus flavomaculatus, pit viper

## Abstract

Introduction: Thrombosis is one of the leading causes of mortality worldwide. Thrombolytic agents are used to reduce this burden. Studies pointed out that certain proteins in the venom of several snake species may have potential thrombolytic properties. *Trimeresurus flavomaculatus*, known as the Philippine pit viper, is found along damp localities in the Philippines. Venoms of closely related species have been shown to exhibit thrombolytic effects in vitro and in vivo. However, no extensive studies yet have been conducted about the thrombolytic effect of *T. flavomaculatus* venom. Thus, this two-phased study aimed to determine the thrombolytic effect of *T. flavomaculatus *venom on human blood and ferric chloride-induced cardiac thrombosis in mice.

Methodology: Phase 1 was done using clot lysis method to measure thrombolytic activity in vitro. Venom dilutions of 3:4, 1:2, 2:3, and 1:0, positive control (streptokinase), and negative control (normal saline solution) were inoculated to different samples of human blood. Phase 2 measured the thrombolytic activity in vivo. Ferric chloride-saturated filter paper was applied over the cardiac wall for the induction of thrombus formation. Venom dilutions of 1:64, 1:16, 1:4, and 1:1, positive control (streptokinase), and negative control (normal saline solution) were then injected through the dorsal tail vein of mice. After 1 hour, the cardiac tissues were excised for histologic examination.

Results: Phase 1 results showed that the venom had significant thrombolytic activity in vitro. Dilutions of 1:0 and 3:4 had no significant differences with streptokinase in vitro. Phase 2 results showed significant lysis in vivo at 1:1, 1:4, and 1:64 venom dilutions.

Conclusion: The results indicated that *T. flavomaculatus* has a potential thrombolytic activity both in vitro and in vivo.

## Introduction

Thrombosis is a key component of arterial thromboembolic diseases, such as myocardial infarction and stroke. Abnormal thrombus formation that can cause obstruction of arteries and veins has three mechanisms namely, endothelial injury, hemodynamic changes, such as blood stasis or increase in turbulence, and hypercoagulability. This mechanism is also known as Virchow’s triad. A thrombus may lodge in vessels of the circulatory system and may cause obstruction leading to hypoxia and accumulation of metabolic products. Tissue may become infarcted when there is total oxygen deprivation [1-4].

Thrombosis is one of the leading disorders affecting a big portion of the human population worldwide. Thromboembolic disorders, such as pulmonary embolism, deep vein thrombosis, ischemic stroke, and heart attack, are responsible for increased morbidity and mortality from vascular diseases in developed countries. New cases of venous thromboembolism in the United States occur in every 100 persons per 100,000 each year and rise exponentially with age. Venous thromboembolism contributes to estimated >500,000 deaths in Europe and up to 300,000 deaths in the United States every year [2,3].

The use of snake venom for medical purposes dates back to ancient times. Recent studies pointed out that several peptides found in the venom of certain snake species may have thrombolytic properties [5,6]. These thrombolytic properties were attributed to the proteases that could degrade fibrin polymer present in thrombi. Proteins such as agkisacutacin [5], saxatilin [6], and atroxase were among the few identified thrombolytic enzymes from venom of certain snake species [7]. Thrombin-like enzymes derived from species of *Trimeresurus gramineus *may be useful as a strong anti-thrombotic agent [8]. Another study on pit viper venom isolated from *Trimeresurus albolabris *and *Trimeresurus macrops *was found to have thrombin-like effect in vitro [5].

*T. flavomaculatus*, commonly known as the Philippine pit viper, is found along streams and damp localities in the Philippine islands. It is endemic in the Philippines and is common at low elevations. This species is listed as least concerned owing to its wide distribution, tolerance to a degree of habitat modification, and large populations occurring in a number of protected and non-protected areas [9]. Though many studies have already been conducted about fibrinolytic enzymes in some snake species, research involving actual in vivo and in vitro experiments using crude venom of *T. flavomaculatus* lack substantial data and further studies are yet to be established.

Objectives

This study aimed to determine the thrombolytic effect of *T. flavomaculatus *venom in vitro in human blood and in vivo in FeCl_3_-induced cardiac thrombosis in Swiss-Webster mice. This study specifically sought to determine whether there are significant differences in the percent clot lysis among samples of clotted human blood treated with the different dilutions of *T. flavomaculatus* venom (undiluted, 3:4, 1:2, and 1:3 dilutions in normal saline solution), negative control (normal saline solution), and positive control (streptokinase) in 90 and 120 minutes.

The in vivo phase of this study aimed to compare the extent of thrombolysis on the FeCl_3_-induced cardiac thrombosis in mice treated with different dilutions of *T. flavomaculatus *venom (1:64, 1:16, 1:4, and 1:1 dilutions in normal saline solution), positive control (streptokinase), and negative control (normal saline solution).

## Materials and methods

This study was conducted in two phases. Procedures were based on a previous study that utilized in vivo and in vitro screening methods to test for thrombolysis [10,11]. Both employed randomized control block design and purposive random sampling. Phase 1 was done in vitro using the clot lysis method. Phase 2 was done in vivo by measuring the size of the thrombus induced by FeCl_3_ after exposure to different treatments.

Venom was extracted using the protocol for snake venom milking published by the World Health Organization. The snake’s head was grasped using the thumb and the index finger just behind the angle of the jaw. Its mouth was then opened exposing the fangs by applying gentle pressure. The fangs were pushed through a plastic membrane and the venom was squeezed out. Samples contaminated with blood were rejected and those viable were preserved at 4°C [12]. Venom samples were then diluted with normal saline solution into 1:0, 3:4, 1:2, and 1:3 dilutions for the in vitro phase and 1:1, 1:4, 1:16, and 1:64 dilutions for the in vivo phase.

In vitro phase

This phase was conducted in a veterinary medicine school laboratory under the supervision of a licensed veterinarian and medical technologist. Fresh blood samples were obtained from research participants who signed the consent form to donate blood samples for the study. Donors were first tested for bleeding time and underwent coagulation assays to ensure that no underlying bleeding disorders were present which may affect the results of the study.

For bleeding time, the Duke method was used. The procedure was done by pricking the fingertip of the participant with a lancet and wiping off the blood with a filter paper every 30 seconds until the bleeding ceases. Normal bleeding time is about 2-5 minutes [13]. For the prothrombin time, the extracted blood was placed in a test tube coated with ethylenediaminetetraacetic acid (EDTA), centrifuged and mixed with calcium and tissue factor. The time it took for the sample to clot was measured optically. For the activated partial thromboplastin time, blood samples were also collected in EDTA tubes. A phospholipid, an activator, and calcium were mixed into plasma sample. The time for clot to form was measured [14].

Blood were drawn from six random donors out of 10 volunteers who passed the screening tests. Ten milliliter of blood was taken from each person, wherein six 1 mL aliquots were obtained and placed in separate test tubes serving as samples. Each sample had six replicates taken from other blood donors. Samples were transferred into labeled pre-weighed sterile test tubes and were allowed to clot in an incubator at 37°C for 45 minutes. The serum was completely removed to isolate the clot. The baseline clot weight was determined by subtracting the weight of the test tube from the weight of the clot-containing test tube. A total of 0.2 mL of snake venom in different dilutions were added to the samples; 0.2 mL of reconstituted streptokinase and normal saline were inoculated to another set of samples and served as positive and negative control, respectively.

The treated samples were incubated at 37°C for 90 minutes for clot lysis to occur. After incubation, the excess fluid was removed and clot weight after treatment was again determined. Difference in weight taken before and after treatment was expressed as percent clot lysis. The same procedure was done to determine the extent of clot lysis after 120 minutes.

In vivo phase

A total of 54 mice were used as subjects. The mice were acclimatized for two weeks, fed with low-fat diet, and maintained with ad libitum water supply. They were divided into three blocks according to how they were housed. A single experimental set-up was composed of six mice randomly picked from one block. Each mouse received a specific treatment. Three replicates were made for each treatment.

The mice were anesthetized via intraperitoneal route with 50 mg/kg Zoletil^ ^(tiletamine HCl 250 mg, zolazepam HCl 250 mg). A left oblique lower cervical incision was made extending through the thorax exposing the common carotid artery, internal jugular vein, and the heart. A 2×4 mm filter paper saturated with 10% (1.2M) FeCl_3_ was applied to the surface of the right ventricle for 20 minutes. After the filter paper was removed, the vessels and the heart were thoroughly washed with normal saline solution.

A total of 0.1 mL of each venom dilution, positive control (streptokinase) and negative control (normal saline solution) were injected through the dorsal tail vein of the mouse subjects. After 1 hour, the mice were euthanized via cervical dislocation under anesthesia. The injured cardiac tissues were isolated and fixed by manual injection of 10% formaldehyde. The heart was excised, immediately immersed in 10% paraformaldehyde, and embedded in paraffin. Carcasses were placed in non-polyvinyl chloride (PVC) sealable, transparent plastic bags and were labeled with identification numbers. Bags were securely sealed and placed inside the carcass container. All carcasses were incinerated [15].

Paraffin blocks containing the organ were sectioned longitudinally into 3 μm slices. Sectioned slices were mounted on glass slides and stained with hematoxylin and eosin. Thrombus size was measured using the longest diameter seen under a light microscope with a calibrated ruler.

Ethical and animal welfare considerations

The study underwent approval from the Unified Biomedical Research Ethics Review Committee of the university for ethics approval. Manipulation of mouse subjects was also approved by the Veterinary Medicine Research Research Review Committee and Animal Welfare Division under the Department of Agriculture. Transport and use of snake venom were approved by the Wildlife Resource Division Protected Animals and Wildlife Bureau under the Department of Environment and Natural Resources.

Statistical processing

For both phases, analysis of variance (ANOVA) was used to determine whether a significant difference occur between the treatment groups. Post-hoc analysis was done using Duncan’s multiple range test (DMRT) with a level of significance of 0.05 to determine which set among the means of the treatment groups differed from the other sets. All statistical computations were computer-processed using Statistical Package for the Social Sciences (SPSS) software version 22.0 (Armonk, NY: IBM Corp.).

## Results

The test for normality using the Shapiro-Wilks test for all phases indicated the data were statistically normal (Table 1). Additionally, using Levene’s test, it was shown that no differences occur between variances across treatment groups in all phases (Table 2). This indicates that the assumption of the ANOVA that variances are equal across groups was met.

**Table 1:** Test of the assumption of normality using the Shapiro-Wilks test at α=0.001 level of significance. NSS: normal saline solution; Sig.: level of significance If p>α, the sample data have been drawn from a normally distributed population.

Phase	Treatment	Shapiro-Wilk		
		Statistic	df	Sig.
In vitro clot lysis at 90 minutes	Venom 1:0	0.987	6	0.981
	Venom 3:4	0.966	6	0.866
	Venom 1:2	0.861	6	0.193
	Venom 1:3	0.901	6	0.377
	NSS (-)	0.883	6	0.284
	Streptokinase (+)	0.834	6	0.116
In vitro clot lysis at 120 minutes	Venom 1:0	0.946	6	0.711
	Venom 3:4	0.817	6	0.084
	Venom 1:2	0.885	6	0.293
	Venom 1:3	0.835	6	0.117
	NSS (-)	0.917	6	0.485
	Streptokinase (+)	0.900	6	0.373
In vivo	Venom 1:1	0.964	3	0.637
	Venom 1:16	0.964	3	0.637
	Venom 1:4	0.964	3	0.637
	Venom 1:64	1.000	3	1.000
	NSS (-)	0.893	3	0.363

**Table 2:** Test of homogeneity of variances using Levene’s test (α=0.05). Sig.: level of significance If p>α, the variances of the populations are homogenous.

Phase	Levene statistic	df1	df2	Sig.
In vitro clot lysis at 90 minutes	3.032	5	30	0.025
In vitro clot lysis at 1200 minutes	3.107	5	30	0.022
In vivo	6.637	5	12	0.003

**Figure 1:**
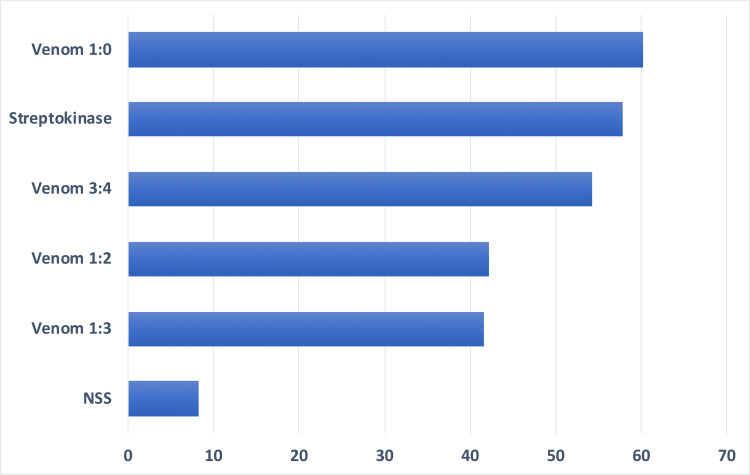
Mean percentage of lysis at 90 minutes of six replicates of various treatment groups. NSS: normal saline solution

Among the treatment variables, the 1:0 dilution of venom yielded the highest percent of clot lysis while the 1:3 dilution yielded the lowest at 90 minutes (Figure 1). The one-way ANOVA showed that there was a highly significant difference between treatment groups, F (5, 30)=18.84, p<0.05, indicating that not all treatment groups showed the same extent of clot lysis in mice at 90 minutes (Table 3). DMRT showed that there were no significant differences in the mean clot lysis using 1:0 and 3:4 venom dilutions and the positive control as treatment. Venom dilution 3:4 also had no significant differences with dilutions 1:3 and 1:2. The negative control had significant differences with all other treatment groups (Table 4).

**Table 3:** ANOVA for the extent of clot lysis in vitro at 90 minutes (α=0.05). Sig.: level of significance

Source	Sum of squares	df	Mean square	F	Sig.
Between groups	11037.822	5	2207.564	18.836	0.0001
Within groups	3516.037	30	117.201	-	-
Total	14553.859	35	-	-	-

**Table 4:** Post-hoc test for clot lysis in vitro at 90 minutes using DMRT (α=0.05). DMRT: Duncan’s multiple range test; NSS: normal saline solution

Treatments	N	Subset		
		1	2	3
NSS (-)	6	8.309	-	-
Venom 1:3	6	-	41.587	-
Venom 1:2	6	-	42.215	-
Venom 3:4	6	-	54.219	54.219
Streptokinase (+)	6	-	-	57.775
Venom 1:0	6	-	-	60.206

**Figure 2:**
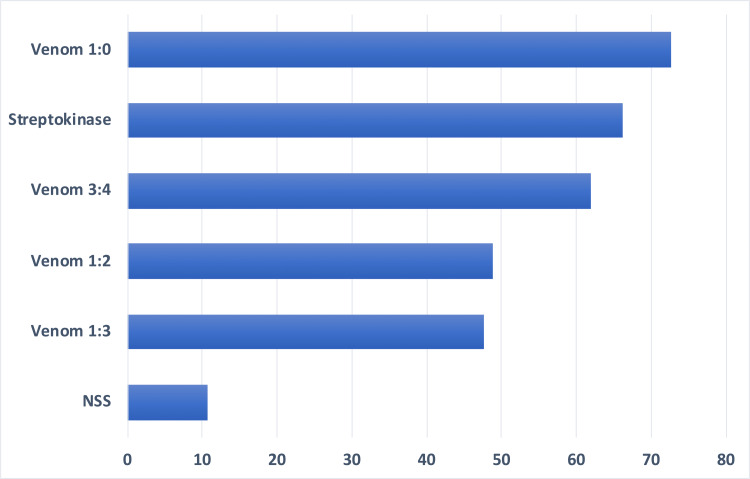
Mean percentage of clot lysis at 120 minutes of six replicates of various treatment groups.

Venom dilution 1:0 yielded the highest percent of clot lysis among the independent treatment variables while the 1:3 venom dilution yielded the lowest at 120 minutes (Figure 2). ANOVA showed that there was a highly significant difference between treatment groups at 120 minutes in vitro, F (5, 30)=23.48 p<0.05 (Table 5). DMRT results showed that there were no significant differences in the mean clot lysis between the positive control and venom dilutions 3:4 and 1:0. Also, venom dilutions 1:2 and 1:3 showed no significant difference from each other. However, there were significant differences between the negative control and all other treatment groups (Table 6).

**Table 5:** ANOVA for the extent of clot lysis in vitro at 120 minutes (α=0.05). Sig.: level of significance

Source	Sum of squares	df	Mean square	F	Sig.
Between groups within groups total	14796.023	5	2959.205	23.481	0.0001
	3780.694	30	126.023	-	-
	18576.717	35	-	-	-

**Table 6:** Post-hoc test for clot lysis in vitro at 120 minutes using DMRT (α=0.05). DMRT: Duncan’s multiple range test; NSS: normal saline solution

Treatments	N	Subset		
		1	2	3
NSS (-)	6	10.663	-	-
Venom 1:3	6	-	47.555	-
Venom 1:2	6	-	48.912	-
Venom 3:4	6	-	-	61.995
Streptokinase (+)	6	-	-	66.240
Venom 1:0	6	-	-	72.683

**Figure 3:**
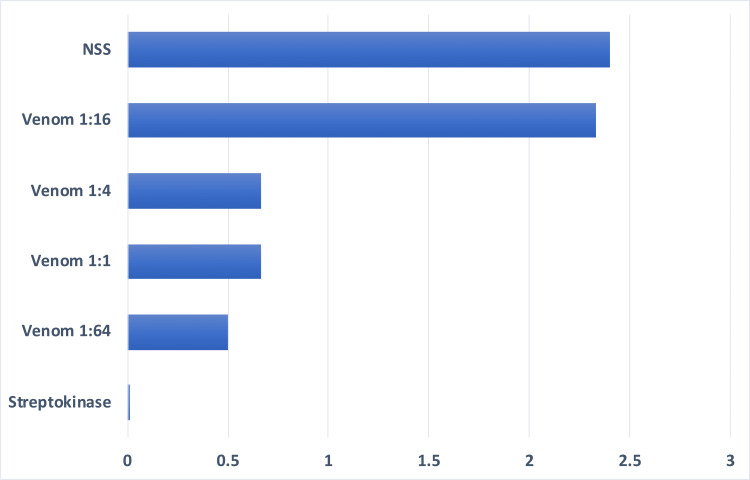
Mean clot length of three replicates of various treatment groups after treatment in vivo. NSS: normal saline solution

In vivo, measurement of the mean clot length for the positive control was least indicating that its therapeutic activity is maximal with virtually total thrombolysis. The negative control showed the least extent of thrombolysis as indicated by having the greatest measurement of the mean clot length. Venom dilution of 1:64 had the lowest measurement among the treatment variables indicating a higher thrombolytic activity at this dilution (Figure 3). ANOVA showed that there was a highly significant difference between treatment groups in vivo, F (5,12)=54.20 p<0.05 (Table 7). DMRT results showed that the extent of thrombolysis in mice treated with venom at 1:64, 1:1, and 1:4 dilutions did not have significant differences with the positive control. Venom dilution 1:16 had significantly different results with the positive control but not with the negative control (Table 8).

**Table 7:** ANOVA for the extent of thrombolysis in vivo (α=0.05). Sig.: level of significance

Source	Sum of squares	df	Mean square	F	Sig.
Between groups	15.469	5	3.094	54.200	0.0001
Within groups	0.685	12	0.057	-	-
Total	16.154	17	-	-	-

**Table 8:** Post-hoc test for thrombolysis in vivo using DMRT (α=0.05). DMRT: Duncan’s multiple range test; NSS: normal saline solution

Treatments	N	Subset	
		1	2
Streptokinase (+)	3	0.000	-
Venom 1:64	3	0.500	-
Venom 1:1	3	0.667	-
Venom 1:4	3	0.667	-
Venom 1:16	3	-	2.333
NSS (-)	3	-	2.400

## Discussion

Both in vitro and in vivo models had significant degree of clot lysis using *T. flavomaculatus* venom. Analysis of data for in vitro clot lysis results at 90 minutes suggests that 1:0 and 3:4 venom dilutions have similar therapeutic activity with streptokinase after 90 minutes.

After 120 minutes, results indicated that the thrombolytic efficacy of streptokinase was comparable with 1:0 and 3:4 venom dilutions of *T. flavomaculatus*. The higher the dilution of venom samples, the larger the extent of clot lysis in vitro.

In phase 2, results of in vivo thrombolysis showed no significant differences among venom dilutions at 1:64, 1:1, and 1:4 and streptokinase. Only the 1:16 venom dilution showed a significant difference with streptokinase but not with normal saline solution. This indicated that in vivo, only 1:16 dilution showed no thrombolytic activity.

There were microscopic evidences that correspond to the process of thrombolysis which support the results. Biopsy specimens from mice treated with normal saline solution showed larger fibrin clots with none to few lytic activity indicating that no pharmacologic action took place but instead physiologic hemostatic process may have occurred. Specimen from mice treated with streptokinase showed intact cardiac tissue without fibrin clot indicating the induced clot has been totally lysed. Those treated with 1:1 venom dilution showed diffuse lysis in several areas around the clot. Treatment with 1:4 dilution showed slight lysis. Specimen treated with 1:64 dilution showed intact fibrin with lytic activity occurring. Specimen treated with 1:16 venom dilution had larger clots but with interspersed areas of lysis. Although statistical analysis showed that 1:16 dilution does not have a thrombolytic activity significantly comparable with streptokinase, it was however evident in the microscopic examination that clot lysis still occurred in mice treated with 1:16 venom dilution but not to a degree sufficient enough to produce a statistically significant effect.

*T. flavomaculatus* venom has shown significant thrombolytic activity which was seen venom of related snake species reported in previous studies. A study about the Asian-lance-headed vipers (genus Trimeresurus) showed that thrombolytic activity is one of the characteristic features of most but not all members of the genus Trimeresurus [16]. Another study about the Malayan pit viper showed that this species has a protein highly specific to fibrinogen, causing anti-coagulation by defibrinogenation [17]. In another study, a protein was also isolated from the snake *T. gramineus *and was reported to have the ability to block the GPIIb/IIIa receptor.

The significant thrombolytic activity in vitro and in vivo of the venom extracted from other Trimeresurus species suggests that similar or another isoform of the thrombolytic enzyme or related peptides could be present in *T. flavomaculatus*.

## Conclusions

Venom extracts of *T. flavomaculatus* in various dilutions has potential thrombolytic effect in vitro and in vivo. Further isolation and characterization of proteins from the venom may help in determining the specific protein responsible for its thrombolytic activity.
